# Circ-ZEB1 promotes PIK3CA expression by silencing miR-199a-3p and affects the proliferation and apoptosis of hepatocellular carcinoma

**DOI:** 10.1186/s12943-022-01529-5

**Published:** 2022-03-11

**Authors:** Weiwei Liu, Lu zheng, Rongguiyi Zhang, Ping Hou, Jiakun Wang, Linquan Wu, Jing Li

**Affiliations:** 1 Department of Hepatobiliary Surgery, the Second Affiliated Hospital of Nanchang University, 1 Mindle Road, Nanchang, Jiangxi 330006 People’s Republic of China; 2 Department of Hepatobiliary Surgery, Xinqiao Hospital, Third Military Medical University, 83 Xinqiao Main Street, Chongqing, 400000 People’s Republic of China

**Keywords:** Circ-ZEB1, miR-199a-3p, PIK3CA, HCC, Proliferation, Apoptosis

## Abstract

**Background:**

Although the prognostic outcomes of liver cancer (LC) cases have improved with the advancement in diagnostic technology and treatment methods, the transferability and recurrence of HCC and the 5-year and 10-year survival rates of patients have remained unsatisfactory. As a result, there is a need for more accurate diagnostic indicators that can detect liver cancer early, effectively improving the prognosis of patients. Whole-genome sequencing (WGS) revealed that circ-ZEB1 and PIK3CA are highly expressed in HCC tissues, whereas miR-199a-3p is significantly downregulated in HCC. Multiple databases search and biological analysis revealed that elevated expression of circ-ZEB1 and PIK3CA was related to poor prognosis of HCC. In vitro and in vivo studies revealed that upregulated levels of PIK3CA and circ-ZEB1 were closely associated with HCC proliferation and apoptosis. Based on these results, we believe that circ-ZEB1 and PIK3CA could be used as biomarkers to diagnose and treat patients with HCC. More importantly, circ-ZEB1 can promotes the expression of PIK3CA by silencing miR-199a-3p and affecting the progression of HCC.

**Methods and results:**

Postoperative specimens from 56 patients with HCC who had not undergone chemotherapy from 2015 to 2018 were collected from the Department of Hepatobiliary Surgery, Second Affiliated Hospital of Nanchang University. WGS revealed differential expression of genes in HCC. Furthermore, RT-qPCR detected the expression of circ-ZEB1, miR-199a-3p, and PIK3CA in HCC tissues. MTT, EdU, and plate cloning experiments were conducted to detect cell proliferation, whereas flow cytometry analysis was used to detect apoptosis. FISH was used to co-localize circ-ZEB1 and miR-199a-3p, and biotin-coupled probe pull-down assay was used to detect the specific binding of circ-ZEB1 and miR-199a-3p. The dual-luciferase report assay detected the association of miR-199a-3p with PIK3CA. Western blotting was used to study the expression of PIK3CA protein. Circ-ZEB1 and PIK3CA were upregulated in HCC and predicted a poor prognosis. MiR-199a-3p showed low expression in HCC, whereas downregulation of circ-ZEB1 reduced HCC cell proliferation and promoted cell apoptosis. MiR-199a-3p blocked the effect of circ-ZEB1 on HCC. Circ-ZEB1 served as a biomarker of HCC. Circ-ZEB1 promoted the expression of PIK3CA by silencing miR-199a-3p to affect the progress of HCC.

**Conclusions:**

Circ-ZEB1 promoted the expression of PIK3CA by depleting miR-199a-3p, thereby affecting HCC proliferation and apoptosis.

## Background

Hepatocellular carcinoma (HCC) is the most common fatal cancer, and it is linked to a number of risk factors, including alcoholism, obesity, and viral infections [1, 2]. Because HCC is characterized by high recurrence and easy metastasis, which are mostly discovered at a very late stage, the prognosis of HCC is poor [3, 4]. Thus, there exists an urgent need for a biomarker for the early diagnosis of HCC.

Increasing evidence has shown that circRNAs are implicated in the development of organisms [5]. CircRNAs participate in transcriptional regulation in the nucleus and compete with mRNA precursors during transcription [6, 7]. Furthermore, these compete with mRNA for the targeted binding site of miRNA and ribosome entry sites, which can translate and express effective proteins [8–10]. Recently, high-throughput sequencing has revealed numerous circRNAs that are closely related to tumor progression. For example, Hong et al. found that circ-CPA4 regulates the proliferation, stem cell characteristics, immune escape, and drug resistance of non-small cell lung cancer (NSCLC) cells via the let-7 miRNA/PD-L1 axis [11, 12]. Similarly, Chen et al. reported that circ-GLI1 interacted with p70S6K2, activated the Hedgehog/Gli1 and Wnt/β-catenin pathways, and upregulated Cyr61, thereby promoting the metastasis of melanoma [13]. Another circRNA, circ-ZEB1, is dysregulated in various tumors and is involved in growth, metastasis, proliferation, apoptosis, and prognosis. Circ-ZEB1 is upregulated in triple-negative breast cancer and is related to its proliferation and apoptosis [14]. In HCC, Gong et al. found that circ-ZEB1 promoted tumor proliferation through the miR-200A-3p-CDK6 axis [15]. Although current studies have reported differences in the expression of circ-ZEB1 in tumors, most of the results are related to molecular phenotype, whereas the underlying mechanism in HCC remains unknown [16, 17]. Moreover, the relationship of circ-ZEB1 with the prognosis of patients with HCC remains to be studied.

MicroRNAs (miRNAs), a class of non-coding RNAs, bind to the 3’UTR of the target mRNA to suppress mRNA translation and decrease their stability [18, 19]. Numerous studies have revealed that the downregulation of miR-199a-3p expression was related to the progression of various tumors [20–22]. For example, circRNA UBAP2 enhances the development of colorectal cancer (CRC) by modulating the miR-199a-3p/VEGFA signal transduction pathway [23]. MiR-199a-3p inhibits epithelial–mesenchymal transition of undifferentiated thyroid cancer cells by targeting SNAIL signals [24]. These studies suggest that miR-199a-3p may function as a tumor suppressor gene, thereby affecting cancer genesis and progression [25–30]. Although our understanding of microRNAs has increased with the continuous advancement of science and technology, gene regulation is complex, and their mutual regulatory relationship still needs to be studied. In addition, PIK3CA encodes class I phosphatidylinositol-3-kinase (PIK3CA), which is the catalytic subunit of PI3Ks and is a member of the family of lipid kinases that specifically phosphorylate the 3-hydroxyl of phosphatidylinositol [31–33]. Moreover, it produces second messengers called inositols. The primary function of the PI3K enzyme is phosphorylation which triggers a series of intracellular signal transmissions by phosphorylation of other proteins [34, 35]. These signals are related to cell activities, including cell proliferation, migration, survival, and the production of new proteins and transport of intracellular material. Studies have found that the activation of PIK3CA mutation promotes adipogenesis of big toe adipose stem cells by upregulating E2F1, and activation of PIK3CA mutation promotes osteogenesis of macro finger bone marrow mesenchymal stem cells [36–38]. Moreover, PIK3CA mutations are closely related to tumor metastasis [39, 40]. According to preliminary data, approximately 27% of breast cancer patients have PIK3CA mutation, more than 20% of endometrial cancer patients have PIK3CA mutation, and approximately 14% of colon cancer patients have PIK3CA mutation [41–44]. Furthermore, Liu et al. found that PIK3CA is regulated by CUL4B and affects bladder cancer metastasis [44, 45]. Based on these studies, we verified the involvement of circ-ZEB1 on the progress of HCC by silencing miR-199a-3p to regulate PIK3CA.

## Results

### Both circ-ZEB1 and PIK3CA showed high expression in HCC

**Fig. 1:**
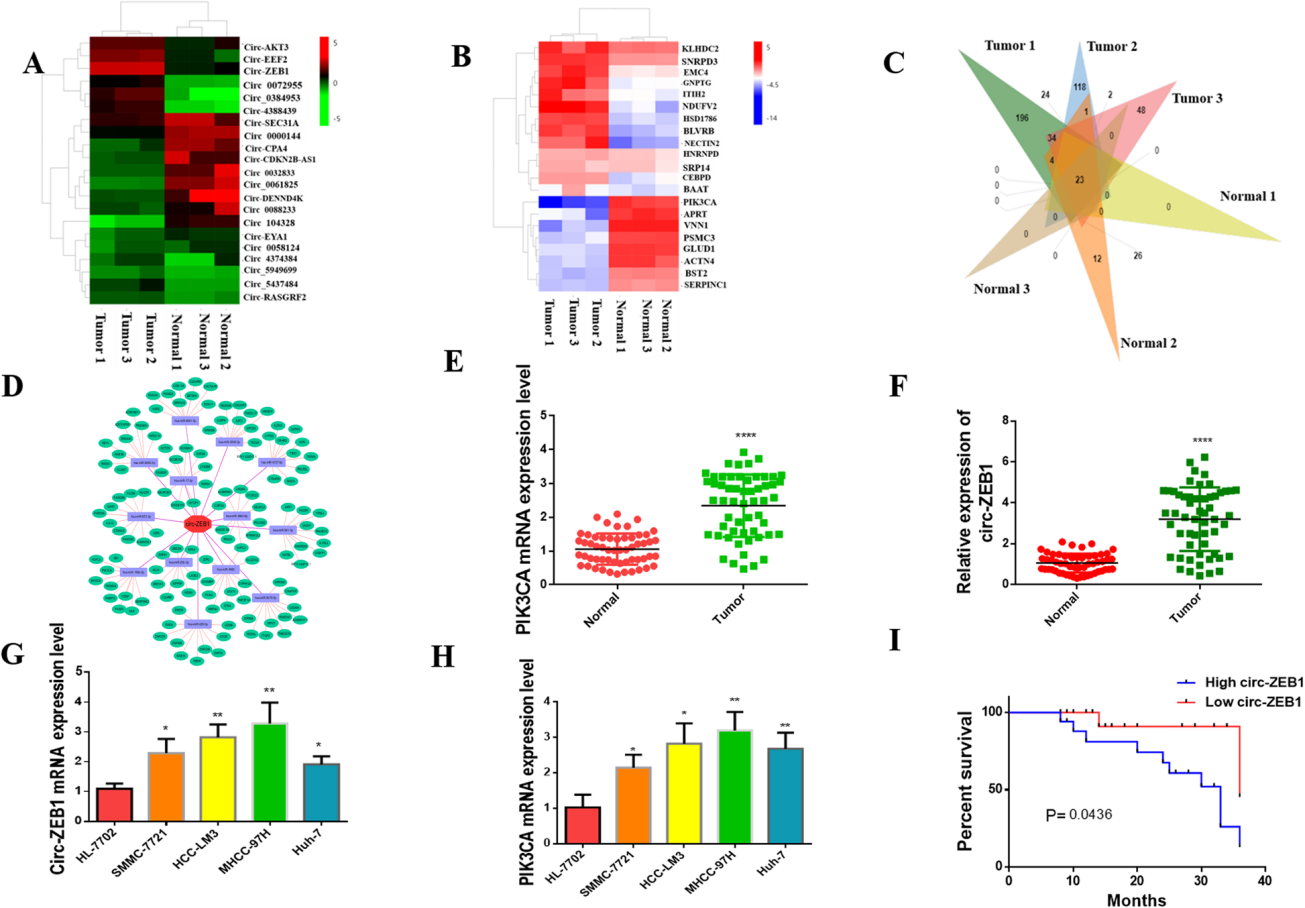
Circ-ZEB1 and PIK3CA are highly expressed in HCC. **A** Whole-genome sequencing revealed that circ-ZEB1 was overexpressed in HCC tissues. **B** Whole-genome sequencing revealed that PIK3CA was overexpressed in HCC tissues. **C** VENN map constructed by whole-genome sequencing. **D** Bioinformatics analysis revealed a relationship between circ-ZEB1 and PIK3CA. **E** RT-qPCR showed that circ-ZEB1 was upregulated in HCC tissues. **F** RT-qPCR showed that PIK3CA was upregulated in HCC tissues. **G** RT-qPCR showed that circ-ZEB1 was upregulated in HCC cells. **H** RT-qPCR showed that PIK3CA was upregulated in HCC cells. **I** The expression of circ-ZEB1 is correlated with the prognosis of HCC

To obtain abnormally expressed genes in HCC tissues, we performed high-throughput sequencing on the newly obtained HCC tissues and adjacent tissues. Sequencing analysis revealed that both circ-ZEB1 and PIK3CA were highly expressed in HCC tissues (Fig. 1A and B). The WGS results of six samples were used to construct a VENN diagram, showing that PIK3CA and circ-ZEB1 levels increased within HCC samples compared with neighboring tissues (Fig. 1C). We simultaneously conducted biological information scientific analysis that revealed a close relationship between circ-ZEB1 and PIK3CA, i.e., PIK3CA was found to be a potential target gene downstream of circ-ZEB1 (Fig. 1D). Following that, we detected HCC using RT-qPCR on 56 pairs of HCC and matched non-carcinoma samples. Our results revealed that not only the expression of circ-ZEB1 and PIK3CA was upregulated in HCC tissues (Fig. 1E and 1F), but circ-ZEB1 was also found in the scatter plot of their expression (Fig. 6C). The level of PIK3CA had a positive correlation.

In addition, the clinical features from 56 cases were collected, including age, gender, hepatitis B virus (HBV) infection or not, tumor size, clinical stage, venous invasion, and distant metastasis (DM). Table 1 summarizes the results. The expression of circ-ZEB1 was markedly correlated with venous invasion, TNM stage, and tumor size. Thus, we conclude that the expression of circ-ZEB1 affected the prognosis of patients with HCC. Moreover, we selected the regular hepatic cell line (HL-7702) and HCC cell lines (Huh-7, HCC-LM3, MHCC-97H, SMMC-7721) to perform RT-qPCR, revealing the expression of circ-ZEB1 and PIK3CA in liver cancer cell lines(Fig. 1G and H). Altogether, both Circ-ZEB1 and PIK3CA are overexpressed in HCC, and the expression of circ-ZEB1 has a good predictive value for the prognosis of patients with HCC (Fig. 1I).

**Table 1:** Clinicopathological characteristics of patients with HCC

**Clinicopathologic characteristics**	**n**	**Overexpression** ***n* = 35**	**Non-overexpression** ***n* = 21**	***p*-value**
**1)** **Age(years)**				*0.193*
** ≤ 51**	*11*	*5*	*6*	
** > 51**	*45*	*30*	*15*	
**2) Sex**				*0.300*
** Male**	*29*	*20*	*9*	
** Femle**	*27*	*15*	*12*	
**3) Tumor size**				***0.044***
** ≤ 5(cm)**	*13*	*7*	*6*	
** > 5(cm)**	*43*	*28*	*15*	
**4) TNM stage**				***0.0002***
** I-II**	*16*	*4*	*12*	
** III-IV**	*40*	*31*	*9*	
**5)Tumor multifocal**				*0.757*
** Absent**	*19*	*11*	*8*	
** Present**	*37*	*24*	*13*	
**6) Venous invasion**				***0.011***
** Absent**	*26*	*11*	*15*	
** Present**	*30*	*24*	*8*	
**7) HBsAg**				*0.053*
** Negative**	*28*	*14*	*14*	
** Positive**	*28*	*21*	*7*	
**8) AFP(ng/ml)**				*0.697*
** ≤ 400**	*15*	*10*	*5*	
** > 400**	*41*	*25*	*16*	
**9) Cirrhosis**				*0.222*
** Absent**	*16*	*8*	*8*	
** Present**	*40*	*27*	*13*	

### Expression of Circ-ZEB1 affects apoptosis in HCC cells

**Fig. 2:**
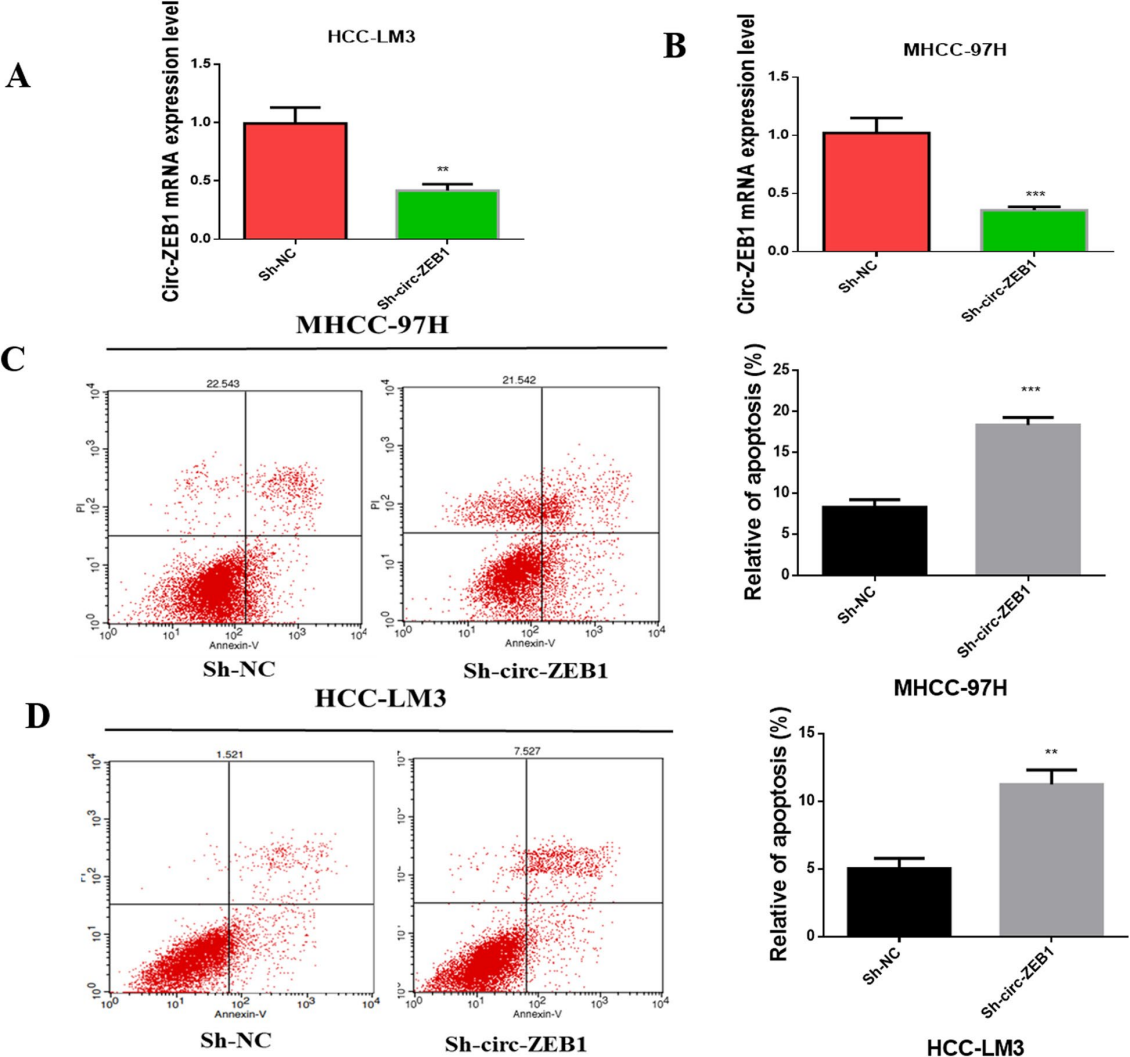
The expression of circ-ZEB1 affects apoptosis in HCC cells. **A**, **B** Changes in the circ-ZEB1 expression in Sh-circ-ZEB1-transfected cells measured by RT-qPCR. **C**, **D** Flow cytometry reflects the changes in apoptosis of cells transfected with Sh-circ-ZEB. The values are presented as mean ± standard deviation (SD). The unpaired *t*-test was used for data analysis. This experiment was performed thrice

To understand how circ-ZEB1 affected HCC cell apoptosis, HCC-LM3 and MHCC-97H cell lines were selected. These cell lines were transfected with knockdown plasmid and control plasmids of circ-ZEB1. Subsequently, RT-qPCR was performed to prove the effectiveness of Sh-circ-ZEB1. Upon transfecting sh-circ-ZEB1 to MHCC-97H and HCC-LM3, the mRNA level of circ-ZEB1 was significantly reduced (Fig. 2A and B). Next, flow cytometry revealed that cells transfected with circ-ZEB1 plasmid significantly enhanced apoptosis(Fig. 2C and D). This result confirmed that downregulating the expression of circ-ZEB1 promotes cell apoptosis.

### Downregulating the expression of circ-ZEB1 reduces the proliferation ability of HCC cells in vitro

**Fig. 3:**
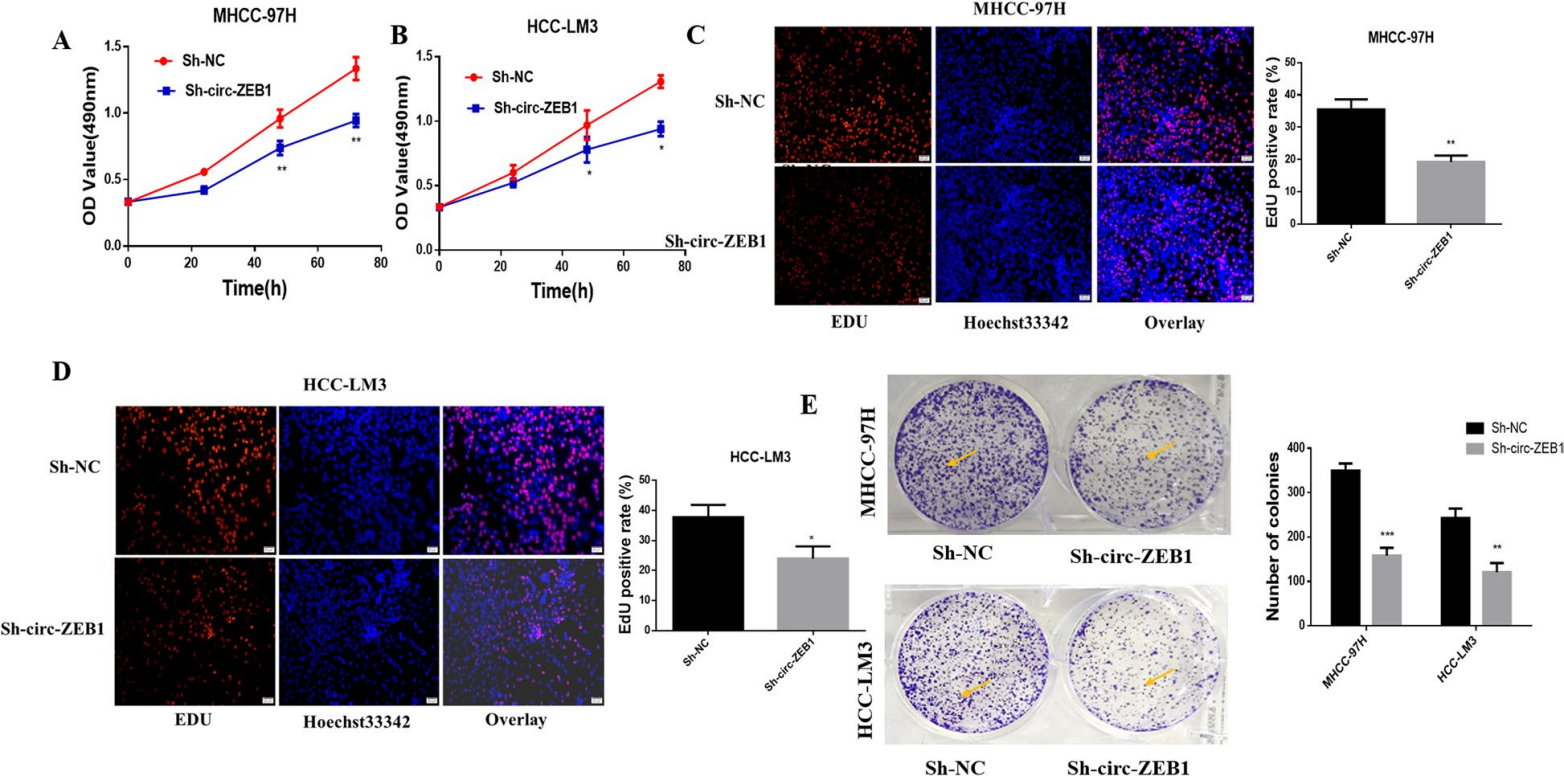
Circ-ZEB1 downregulation reduces the proliferation ability of HCC cells in vitro. **A** The MTT assay was used to detect the changes in the MHCC-97H activity after the downregulation of circ-ZEB1. **B** The MTT assay was used to detect the changes in the HCC-LM3 activity after the downregulation of circ-ZEB1. **C** EdU assay reveals the changes in MHCC-97H cell proliferation after downregulation of circ-ZEB1. **D** EdU assay reveals the changes in the proliferation of HCC-LM3 cells after the downregulation of circ-ZEB1. **E** Plate cloning experiment was used to detect cell proliferation changes after transfection of cells with Sh-circ-ZEB1. ^*^P < 0.05 compared with the Sh-NC group. All values are presented as mean ± standard deviation (SD). Comparisons between the groups were analyzed by unpaired *t*-test, whereas repeated measures analysis of variance (ANOVA) was used to compare several groups. This experiment was performed thrice

To evaluate how circ-ZEB1 affected cell proliferation, we performed MTT, plate cloning, and EdU assays. First, HCC cells were transfected with Sh-circ-ZEB1 and Sh-NC, and subsequently, cells were subjected to MTT, which revealed that Sh-circ-ZEB1 transfected cells had reduced proliferation compared with control cells(Fig. 3A and B). The EdU assay indicated that the downregulation of circ-ZEB1 reduced cell proliferation(Fig. 3C and D). In the plate cloning experiment, cells with downregulated circ-ZEB1 proliferated at a significantly lower rate than controls (Fig. 3E). Therefore, the expression of circ-ZEB1 was markedly associated with cell proliferation, whereas its downregulation suppressed the proliferation of HCC cells.

### Circ-ZEB1 specifically binds to miR-199a-3p

**Fig. 4:**
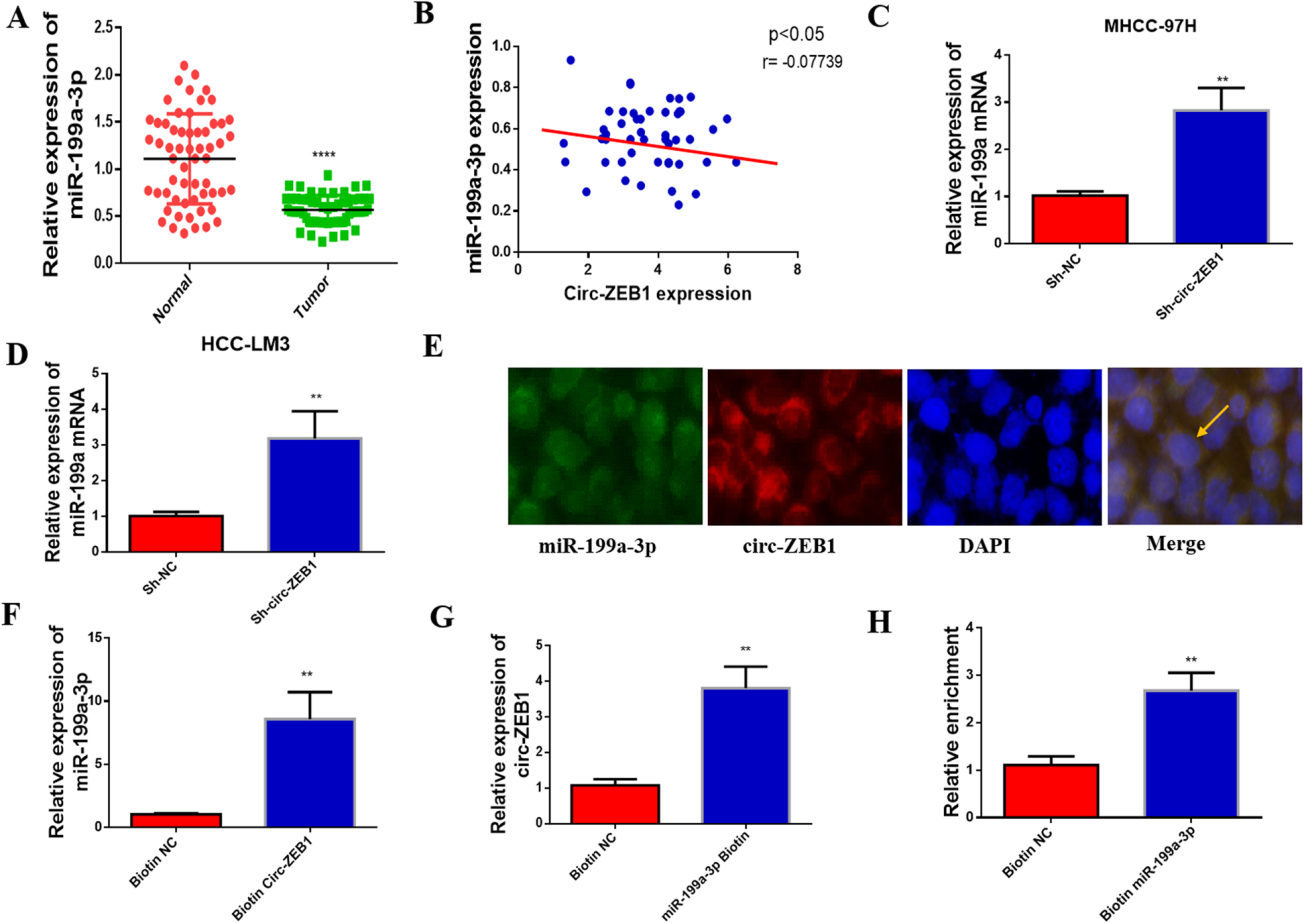
Circ-ZEB1 binds to miR-199a-3p. **A** MiR-199a-3p levels within HCC and matched non-carcinoma samples were measured by RT-qPCR. **B** Circ-ZEB1 expression was positively correlated with miR-199a-3p expression in HCC. **C**, **D** Changes in the expression of miR-199a-3p after downregulation of circ-ZEB1 were measured by RT-qPCR. **E** The FISH assay revealed the co-localization of circ-ZEB1 with miR-199a-3p (^*^400). **F** Circ-ZEB1 enriched with a specific probe detected by RT-qPCR. **G** The enriched miR-199a-3p RT-qPCR detected circ-ZEB1-specific probe. **H** Circ-ZEB1 expression was trapped using the biotin-conjugated miR-199a-3p and measured by RT-qPCR. ^*^P < 0.05 versus biotin Sh-NC group. All values are presented as mean ± standard deviation (SD). Comparisons between the groups were analyzed by unpaired *t*-test. This experiment was performed thrice

For understanding the association of circ-ZEB1 with miR-199a-3p, this study measured the miR-199a-3p expression within HCC samples. Firstly, miR-199a-3p expression decreased within the HCC tissues (Fig. 4A). Simultaneously, we found that circ-ZEB1 expression was negatively correlated with miR-199a-3p level (Fig. 4B). Therefore, transfection of cells with Sh-circ-ZEB1 reversely increased the miR-199a-3p levels (Fig. 4C and D). To further study the relationship between circ-ZEB1 and miR-199a-3p, the circ-ZEB1-specific probe was used to delete or overexpress circ-ZEB1 (Fig. 4F). Thus, the overexpression of miR-199a-3p and circ-ZEB1 was detected in beads-knocked down biotin-labeled RNA complex, proving the direct binding of circ-ZEB1 to miR-199a-3p (Fig. 4G). FISH analysis and biotin-coupled miRNA capture confirmed that circ-ZEB1 was bound to miR-199a-3p (Fig. 4E and H). Higher expression of circ-ZEB1 was detected within biotin-labeled miR-199a-3p, indicating that miR-199a-3p can bind to circ-ZEB1.

### Silencing miR-199a-3p inhibits the regulation of circ-ZEB1 on the proliferation and apoptosis of HCC cells

**Fig. 5:**
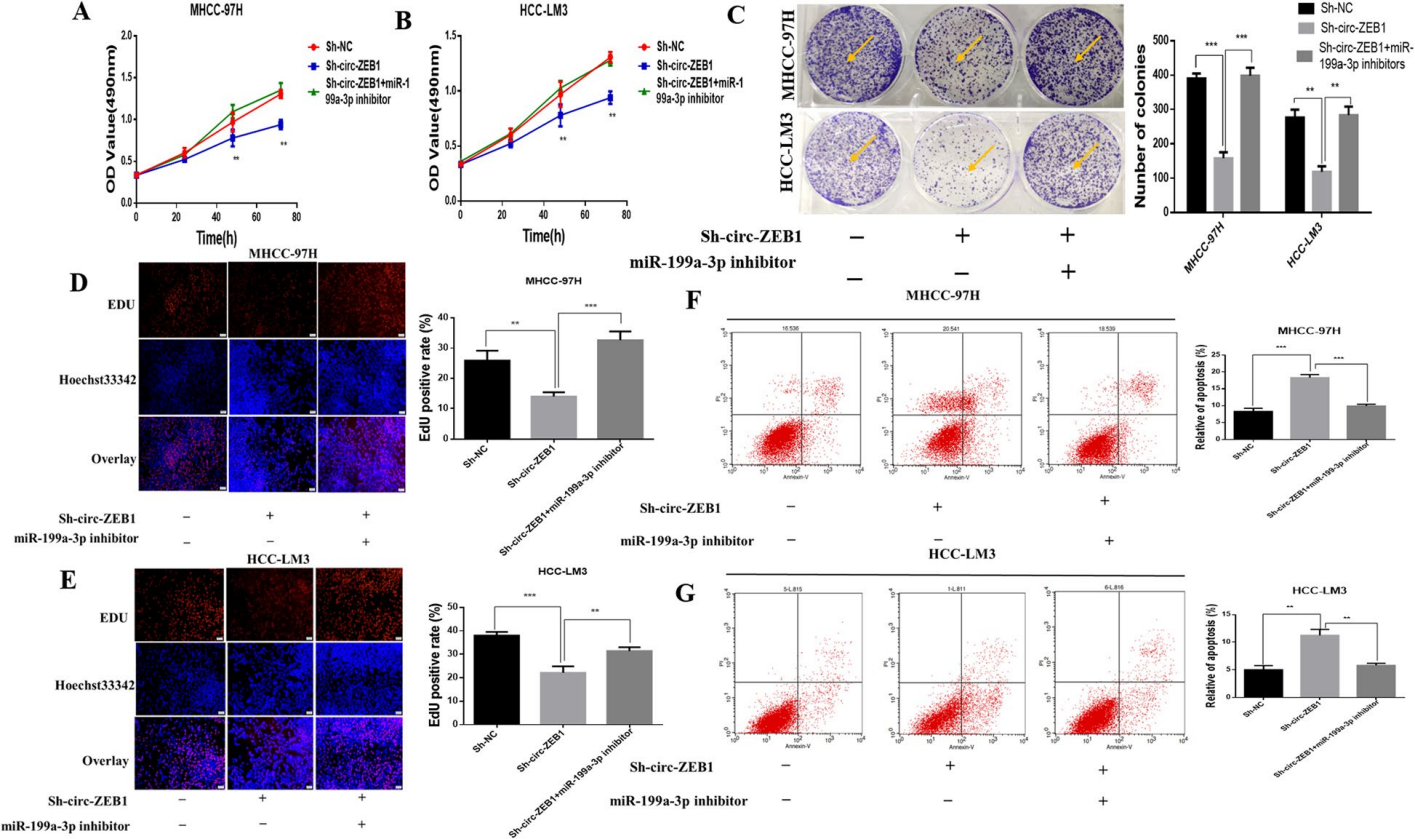
Silencing of miR-199a-3p inhibits the regulation of HCC cell proliferation and apoptosis by circ-ZEB1. **A** The MTT assay was used to detect the activity change in MHCC-97H cells after simultaneous downregulation of circ-ZEB1 and miR-199a-3p. **B** The MTT assay was used to detect the change in the HCC-LM3 activity after simultaneous downregulation of circ-ZEB1 and miR-199a-3p. **C** The plate cloning experiment reflects the changes in the proliferation ability of MHCC-97H and HCC-LM3 after simultaneous transfection of cells with Sh-circ-ZEB1 and miR-199a-3p inhibitors. **D** Changes in the proliferation of MHCC-97H cells after simultaneous transfection of cells with Sh-circ-ZEB1 and miR-199a-3p inhibitors detected by EdU assay. **E** Changes in the proliferation of HCC-LM3 cells after co-transfection with Sh-circ-ZEB1 and miR-199a-3p inhibitors and detected using EdU assay. **F** Flow cytometry revealed apoptotic changes in MHCC-97H cells after simultaneous downregulation of circ-ZEB1 and miR-199a-3p. **G** Flow cytometry revealed apoptotic changes in HCC-LM3 cells after simultaneous downregulation of circ-ZEB1 and miR-199a-3p

Previous results confirmed that circ-ZEB1 and miR-199a-3p-3p have specific binding sites, and circ-ZEB1 can negatively regulate miR-199a-3p. To determine whether the presence of miR-199a-3p inhibited the effect of circ-ZEB1 on HCC, we designed a retrospective experiment, where both circ-ZEB1 and miR-199a-3p were downregulated. The MTT assay showed that downregulation of circ-ZEB1 reduced cell proliferation; however, simultaneous downregulation of miR-199a-3p restored cell proliferation (Fig. 5A and B). Plate cloning experiments revealed that when circ-ZEB1 and miR-199a were downregulated simultaneously, the proliferation ability of cells improved compared to only downregulating circ-ZEB1 (Fig. 5C). The EdU assay revealed that when the expression of miR-199a-3p and Circ-ZEB1 decreased simultaneously, circ-ZEB1 downregulation markedly enhanced cell proliferation (Fig. 5D and E). Apoptosis experiments revealed that inhibiting circ-ZEB1 promoted apoptosis; however, simultaneous inhibition of miR-199a-3p reduced the apoptosis ability (Fig. 5F and G). Therefore, we concluded that circ-ZEB1 affected HCC cell proliferation and apoptosis via miR-199a-3p; however, miR-199a-3p can simultaneously prevent the effect of circ-ZEB1 on HCC.

### Circ-ZEB1 positively regulates the target gene *PIK3CA* of miR-199a-3p

**Fig. 6:**
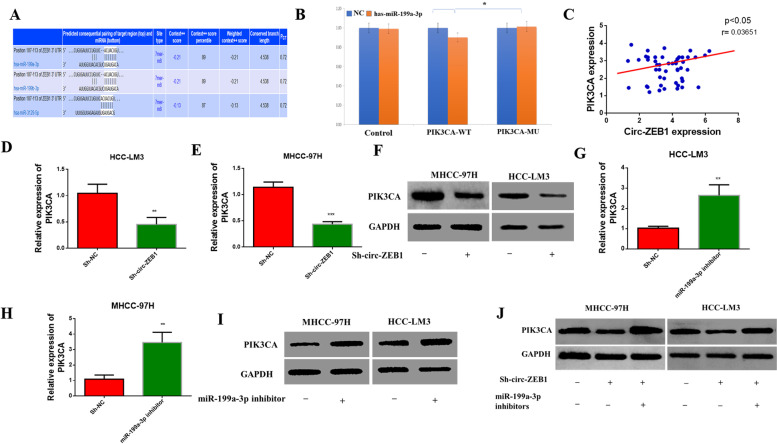
Circ-ZEB1 positively regulates the target gene *PIK3CA* of miR-199a-3p. **A** Bioinformatics analysis shows that circ-ZEB1 and PIK3CA have binding sites. **B** The dual-luciferase reporter assay reveals *PIK3CA* as the target gene of circ-ZEB1. **C** Correlation analysis between circ-ZEB1 and PIK3CA. **D**, **E** Changes in PIK3CA mRNA expression in circ-ZEB1 knockdown cells detected by RT-qPCR. **F** Changes in the protein expression of PIK3CA in circ-ZEB1 knockdown cells measured by western blotting. **G**, **H** Changes in the mRNA expression of PIK3CA in miR-199a-3p inhibitor-transfected cells measured by RT-qPCR. **I** Changes in the protein expression of PIK3CA in miR-199a-3p inhibitor-transfected cells measured by western blotting. **J** The expression change in PIK3CA protein level after simultaneously knocking down circ-ZEB1 and downregulating miR-199a-3p. Statistical analysis was performed by one-way analysis of variance (ANOVA) or repeated-measures ANOVA. Pearson’s correlation analysis was used to correlate the two groups. This experiment was performed thrice

We next studied whether there existed a connection between miR-199a-3p and PIK3CA. First, a UTR-binding site was predicted between PIK3CA and miR-199a-3p-3p based on the information on the website (www.microRNA.org) (Fig. 6A) and that *PIK3CA* might serve as the miR-199a-3p target gene. A dual-luciferase reported assay for miR-199a-3p-3p and PIK3CA revealed a binding site, and *PIK3CA* was identified as the miR-199a-3p-3p target gene (Fig. 6B). We found that circ-ZEB1 and PIK3CA expressed a positive linear relationship by running RT-qPCR (Fig. 6C). Next, downregulating circ-ZEB1, PIK3CA mRNA, and protein expression decreased on average (Fig. 6D–F), whereas downregulating miR-199a-3p exerted an opposite effect (Fig. 6G–I). Finally, simultaneous downregulation of circ-ZEB1 and miR-199a-3p restored PIK3CA protein expression (Fig. 6J). Based on these results, circ-ZEB1 positively regulated the target gene *PIK3CA* of miR-199a-3p.

### Downregulating circ-ZEB1 inhibits tumor growth in vivo

**Fig. 7:**
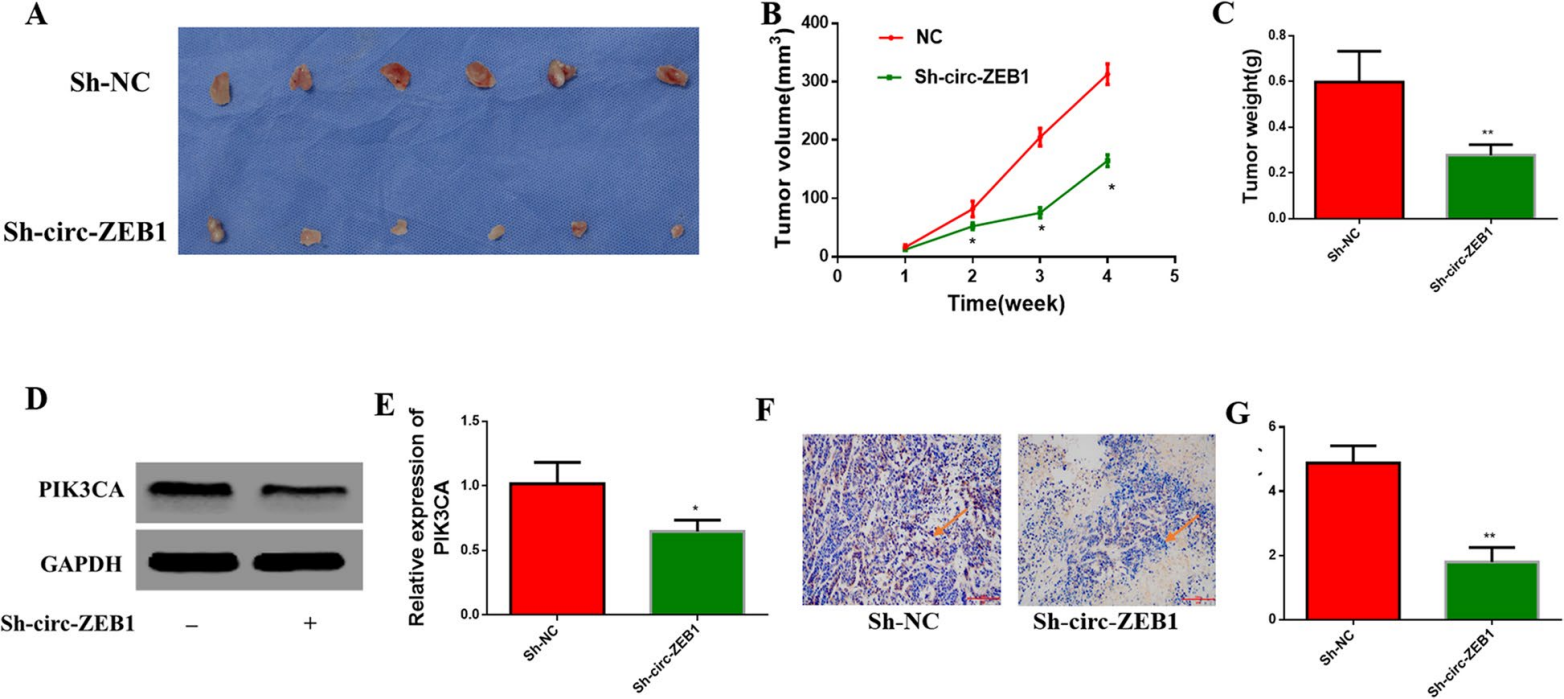
Downregulation of circ-ZEB1 inhibits the formation of xenograft tumors in nude mice. **A**–**C** Xenograft formation, tumor volume, and tumor weight in nude mice, respectively. **D**, **E** Western blotting for PIK3CA in nude mice treated with HCC-LM3 cells transfected with Sh-circ-ZEB1 and NC. **F**, **G** IHC (^*^400) detects the positive rate of PIK3CA in nude mice treated with HCC-LM3 cells transfected with Sh-circ-ZEB1 and NC. ^*^P < 0.05 versus the NC group. All data are presented as mean ± standard deviation (SD). The unpaired *t*-test was used to analyze the comparison between the two groups

To observe the effect of circ-ZEB1 on tumors in vivo, we injected the HCC-LM3 cells transfected with Sh-circ-ZEB1 and Sh-NC into two groups of nude mice. We recorded weekly changes in the tumor volume in nude mice, which revealed that the growth rate and size of tumors in the group of mice transfected with Sh-circ-ZEB1 were lower than those in the other group (Fig. 7A, B and C). The collected tumor samples were used to perform RT-qPCR and western blotting, revealing markedly decreased PIK3CA mRNA and protein expression in the experimental group with circ-ZEB1 downregulation as compared with that in the Sh-NC group (Fig. 7D and 7E). Moreover, we verified the reduced expression of PIK3CA in the experimental group by immunohistochemistry (Fig. 7F and 7G). Based on these results, we conclude that silencing circ-ZEB1 can inhibit the growth of HCC in nude mice.

## Discussion

Hepatocellular carcinoma ranks fifth among the frequently observed cancers worldwide and second place among the leading causes of cancer-associated mortality [46, 47]. Its therapeutic effect is generally poor due to high heterogeneity, easy metastasis, and high recurrence [4]. In the present study, we found a target molecule for HCC treatment, which could be used as a potential candidate for diagnosing and treating HCC.

Recently, whole-genome sequencing has revealed abnormal expression of several circRNAs in various tumors. Moreover, circRNAs have crucial functions in the genesis and progression of different cancers [48–50]. For instance, Zhao et al. found that propofol blocked the tumor and aerobic glycolysis of lung cancer cells by regulating the TADA2A/miR-455–3p/FOXM1 axis [51]. Similarly, Li et al. reported that circTADA2A inhibited the activation of lung fibroblasts through miR-526b/Cav1 [52]. Furthermore, it inhibited the proliferation of lung fibroblasts through miR-203/Cav2, thereby preventing the excessive deposition of ECM and reducing idiopathic pulmonary fibrosis (IPF) [53]. A recent study discovered that the intestinal microflora regulated the level of corresponding miRNAs by influencing circRNA expression, demonstrating that circRNA plays an important role [5, 10, 54, 55]. Our in vitro and in vivo experiments suggested that the expression of circ-ZEB1 increased in HCC, which was tightly associated with the prognosis. Downregulation of circ-ZEB1 significantly reduced the proliferation ability of HCC cells and promoted cell apoptosis. Circ-ZEB1 may serve as a diagnostic marker and therapeutic target for HCC.

MicroRNA is a non-coding RNA discovered in the past few decades. An extensive literature review revealed that most microRNAs in tumors function as tumor suppressor genes, and most of them exhibit reduced expression [56–59]. For example, Liang et al. demonstrated that miR-29a exerted a tumor suppressor effect through IFITM3 in HCC [60]. Similarly, Ai J et al. reported that miR-181c affected the progression of HCC through NCAPG [61]. However, several recent studies suggest that microRNAs can be regulated by lncRNAs and circRNAs [62–64]. For example, Almairac et al. showed that ERK-mediated deletion of miR-199a-3p-3p and induction of EGR1 act as “trigger switches “ for the dedifferentiation of GBM cells into NANOG-and OCT4-positive cells [65]. Zhang et al. found that the hypoxia-mediated lncRNA-NEAT1 maintained HCC development via regulating the miR-199a-3p-3p/UCK2 axis [30]. In contrast, the low expression of miR-199a-3p is detected in diverse cancers. The literature reports that circ-ZEB1 specifically binds miR-199a-3p-3p, and a negative regulatory relationship exists between the two. Moreover, we observed specific binding sites of these two molecules through FISH co-localization and biotin-coupled probe pull-down assays. We next downregulated circ-ZEB1, suggesting the upregulated miR-199a-3p levels and verifying that miR-199a-3p blocked the cancer-promoting effect of circ-ZEB1.

Phosphatidylinositol 3-kinase (PI3K) is tightly associated with the growth and development of the body [66]. For example, somatic mutations in PIK3CA result in systemic lymphatic abnormalities [67]. The mutation in PIK3CA is related to the adipogenesis of adipose stem cells [68]. Subsequent research has revealed that PIK3CA mutations are linked to the prognosis of various tumors [38]. Herberts et al. found that activating mutations in AKT1 and PIK3CA are responsible for metastatic castration resistance to prostate cancer [69]. Luo et al. found that the mutation in PIK3CA affected the prognosis of colorectal cancer [70]. A recent study reported that a PIK3CA mutation resulted in a difference in the prognosis of HPV-related OPSCC patients receiving de-enhanced radiotherapy and chemotherapy [71]. Increasing evidence indicates that PIK3CA mutations can affect tumor progression [34, 38]. We found that PIK3CA has the potential as a diagnostic and prognostic marker. Furthermore, dual-luciferase reported assay identified *PIK3CA* as the miR-199a-3p downstream target, which was negatively regulated by the latter. As a result, miR-199a-3p can regulate *PIK3CA* as an oncogene, and mutant PIK3CA is frequently found in HCC. Our previous studies reported that circ-ZEB1 and PIK3CA were overexpressed in HCC tissues and cells. The linear statistical analysis revealed that circ-ZEB1 was positively correlated with PIK3CA. Downregulation of circ-ZEB1 markedly reduced the expression of PIK3CA mRNA and protein expression. Therefore, we infer that circ-ZEB1 positively regulated *PIK3CA* and miR-199a-3p targeted circ-ZEB1 to prevent it from inducing *PIK3CA* expression.

We used whole-genome sequencing and searched multiple databases to find that circ-ZEB1 and PIK3CA are overexpressed in various tumors, with reduced expression of miR-199a-3p. The RT-qPCR analysis confirmed the expression of these three molecules in HCC. The correlation between the three molecules was found through statistical data analysis, biotin coupling, FISH probe detection, and dual-luciferase reporter assay. Finally, we validated our results by in vitro functional and in vivo animal experiments. In summary, circ-ZEB1 affected the proliferation and apoptosis of HCC. More importantly, we showed that the silencing of circ-ZEB1 intervened with the progression of HCC by targeting miR-199a-3p to inhibit *PIK3CA* expression. Although we found a mutual adjustment relationship between the three molecules, all experimental results are based on in vitro cell experiments and animal experiments, and the effects of clinical experiments require further validation.

## Conclusion

**Fig. 8:**
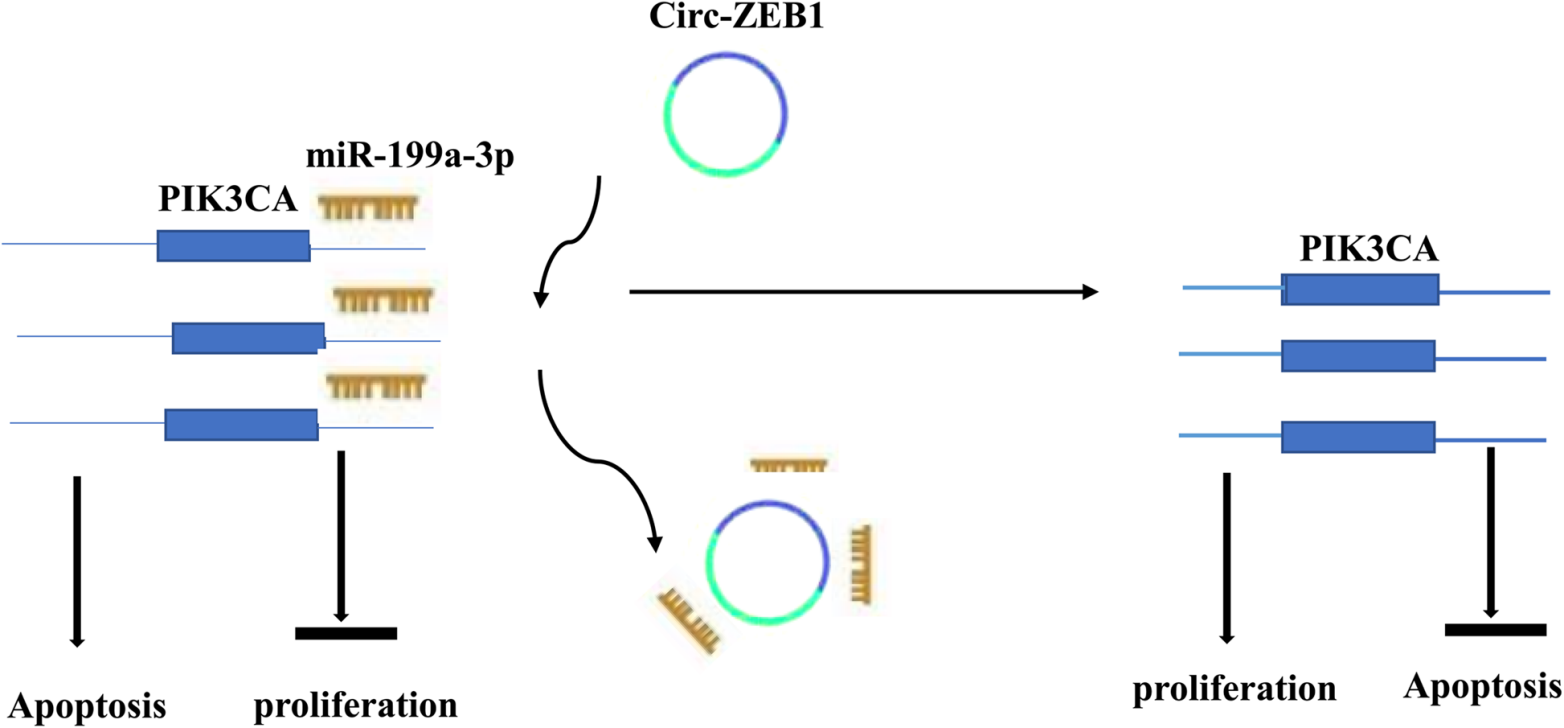
Schematic diagram of the function of Circ-ZEB1. This represents that the combination of circ-ZEB1 targeting and miR-199a-3p affects the expression of PIK3CA, which in turn alters the proliferation and apoptosis of the HCC cells. When circ-ZEB1 is downregulated, miR-199a-3p bound to PIK3CA increases, which will promote cell apoptosis and inhibit cell proliferation

In conclusion, our findings show that circ-ZEB1 inhibits HCC cell apoptosis while promoting cell proliferation. Circ-ZEB1 can be used as a biomarker to diagnose and treat HCC. More importantly, silencing of circ-ZEB1 intervenes with the progression of HCC by targeting miR-199a-3p to inhibit PIK3CA expression (Fig. 8).

## Materials and methods

### Ethics Statement

The present study was approved by the Ethics Committee of Second Affiliated Hospital of Nanchang University. According to the Declaration of Helsinki, all participants signed an informed consent form before data collection. In addition, animal experiments in this study were approved in writing by the Institutional Animal Research Committee of the Second Affiliated Hospital of Nanchang University.

### Microarray-based circRNA expression profile

HCC-related circRNA expression profiling data were obtained against the Gene Expression Comprehensive (GEO) database (https://www.ncbi.nlm.nih.gov/geo/). Thereafter, the obtained data were preprocessed using the R Affy package. Afterward, differentially expressed circRNAs (DE circRNAs) were selected at the thresholds of | log2FC |> 1.5 and circRNA (ad.P.Val < 0.05) and verified using the R Limma package. The adjusted p-value was presented using adjust.P.Val. Later, the acquired circRNAs were plotted onto a graph. Moreover, the candidate regulatory mechanism of circ-ZEB1 was speculated using the Cytoscape tool (https://cytoscape.org/). In addition, the circRNA-regulated gene expression, along with survival curves, was analyzed using The Cancer Genome Atlas (TCGA) database (http://ualcan.path.uab.EdU/index.html).

### Patients and samples

The liver cancer tissues and para-cancerous tissues from patients with HCC were collected from 2015 to 2018 from the Department of Hepatobiliary Surgery, Second Affiliated Hospital of Nanchang University. No medication was received before the sample was collected. The harvested samples were soaked in liquid nitrogen. The follow-up time was three years.

### Cell culture

HCC cell lines (SMMC-7721, HCC-LM3, MHCC-97H, and Huh-7) and the normal hepatic cell line HL-7702 were incubated in DMEM (Thermo Fisher Scientific) and incubated with 10% FBS (Thermo Fisher Scientific, Inc.) DMEM medium, supplemented with 100 µg/mL streptomycin and 100 U/mL penicillin, followed by incubation at 37 °C, saturated humidity, and 5% air condition. Cells growing in the log phase were used for all experiments.

### RNA extraction and determination

The TRIzol kit (Shanghai Harin Biotechnology Co., Ltd., Shanghai, China) was used to extract total cellular RNA. The reverse transcription kit was used to synthesize cDNA (Takara Biotechnology Co., Ltd., Dalian, China). Next, the SYBR Premix Ex TaqTM II kit for qRT-RCR was used. GAPDH and U6 were used as internal references using the following calculation formula: 2^–ΔΔCt^ method was used to calculate the relationship between the gene expression ratios between the two groups, the formula used was as follows: MMC_t_ = MC_t_ experimental group and MC_t_ control group, which is 2^–ΔΔCt^. All primers were purchased from Guangzhou Ruibo Biological Company, and the sequence is provided in Table 2.

**Table 2:** Primer sequence

Target Gene	Primer(5’-3’)
Circ-ZEB1	F:5’-TGTGGGGTGTGAGAACTTGA-3’ R: 5’-ATGCTGCTTTGACAGGGTTT-3’
miR-199a-3p	F:5’-AGCTTCTGGAGATCCTGCTCC-3’ R:5’-TCCCTTGCCCAGTCTAACCAA-3’
PIK3CA	F:5’ -CATGCATTGTTTTGCACCCC-3’ R: 5’-ATGGAAGACGGGAGATTCACAT-3’
GAPDH	F:: 5’-TATGATGATATCAAGAGGGTAGT-3’ R: 5’-TGTATCCAAACTCATTGTCATAC-3
U6	F: 5’ -CTCGCTTCGGCAGCACA-3’ R:5’ -AACGCTTCACGAATTTGCGT-3’

### Western blotting

We used the Bio-Rad DC protein detection kit (Guangzhou Ewell Biotechnology Co., Ltd., China) to determine the protein content. Afterward, the obtained proteins were separated through SDS/PAGE, then transferred onto the PVDF membranes. After that, the TBST containing 5% skimmed milk was used to block the membranes, followed by 2 h of gentle shaking to avoid non-specific binding. Later, primary antibodies shown below were used to incubate the membranes, including rat anti-human glycerol triphosphate dehydrogenase (1:5000, ab8245; GAPDH; Abcam Inc., Cambridge, UK) and rabbit anti-human PIK3CA (1:1000, ab183957; Abcam Inc., Cambridge, UK) at 4 °C overnight. Next, the membranes were washed by TBST, followed by further incubation with goat anti-rabbit IgG secondary antibody (1:20,000, ab6721; Abcam Inc.) for 1 h. Next, the membranes were rinsed thrice with TBST.

### Cell transfection

HCC-LM3 and MHCC-97H were used as experimental cell lines. When cells were grown to a concentration of 70%, the interference fragment of circ-ZEB1 (Sh-circ-ZEB1) and miR-199a-3p inhibitor were transferred into the cells using Lif3000 according to the manufacturer’s instructions (Guangzhou Ruibo Biological Co., Ltd.). Next, a serum-free medium was added, and cells were incubated for 6 h, following which the cells were incubated in a medium containing 10% serum and cultured for another one to two days.

### 3-[4,5-Dimethylthiazol-2-yl]-2,5-diphenyltetrazolium bromide (MTT) determination

First, cells were inoculated into a 96-well plate, and each well was incubated with 20 µL of MTT solution (Shanghai Fortune Technology Co., Ltd., China) for 4 h and then centrifuged. After removing the supernatant, 100 µL of dimethyl sulfoxide was added to each well, followed by 10 min of gentle shaking using the micro shaker. A microplate reader was used to measure the optical density at 490 nm when all purple crystals were completely dissolved.

### Plate cloning experiment

The clone formation rate indicated the proliferation capacity and cell population dependency. Cells in the logarithmic phase were harvested, digested using 0.25% trypsin, added into the single cells, and cultured in a 6-well plate. The culturing was stopped when visible clones were observed in the Petri dish. After discarding the supernatant, cells were rinsed carefully twice with PBS. Next, 5 mL of 4% paraformaldehyde was used to fix the cells for 15 min. Later, the fixation solution was removed, a suitable volume of GIMSA was added, and the dye solution was added to the stain for 10 to 30 min. Running water was used to clear the dye solution carefully, followed by air drying. Next, the plate was turned upside down, and a grid-containing transparent film was superimposed onto the plate. The number of clones was calculated macroscopically; alternatively, clones containing at least 10 cells were calculated using the microscope (low power). Finally, the rate of clone formation was determined.

### Determination of 5-ethynyl-2ʹ-deoxyuridine

Cell proliferation was measured by the proliferation assay. In brief, 50 µM EdU (0.1 mL, RiboBio Biotechnology, Guangzhou, China) was added to every well containing 500 mL of medium for 2 h. After fixing with 4% polyoxymethylene dissolved in PBS under ambient temperature for 30 min, cells were cultured for 30 min using Hoechst 33,342 and Apollo staining solution. Later, five fields of view (FOVs) were randomly selected for fluorescence microscopy using the Olympus microscope (Tokyo, Japan) for analyzing cell proliferation. The nuclei were stained blue with Hoechst 33,342; EdU in proliferating cells was stained with red Apollo. Next, the proliferating cell proportion was determined. Later, fluorescence microscopy was used to monitor the stained cells, and images were acquired by a camera. Finally, the total cell and proliferating cell numbers were counted using Image J.

### Determination of apoptosis

The treated cells were incubated for 48 h. Next, the cells were eluted and collected in a flow cytometry tube. According to the flow cytometry instructions, the cells were stained for 30 min (Guangzhou Ruibo Biological Co., Ltd.).

### Biotin-coupled probe pull-down assay

A biotin-conjugated probe of circ-ZEB1 with miR-199a-3p-3p-binding region was designed. The pre-chilled PBS was used to wash a total of 1 × 10^7^ cells, followed by immersion in the lysis buffer, and combined with 3 ug biotin The probe was incubated together for 2 h. Afterward, the streptavidin-loaded magnetic beads (Thermo Fisher Scientific; Waltham, MA, USA); Life Technologies Corporation (Gaithersburg, MD, USA) were used to incubate cell lysate for 4 h to extract the biotin-labeled RNA complex. After washing the magnetic beads with lysis buffer five times, TRIzol reagent was used to separate the bound miRNA from the complex, followed by RT-PCR.

### Biotin-coupled miRNA capture

Altogether, 2 × 10^6^ cells were transfected with 50% biotinylated miRNA mimic (50 mL; GenePharma, Shanghai, China) for 24 h. Afterward, the cells were collected, washed with PBS, and lysed in the lysis buffer. Next, cells were sealed with 50 mL of streptavidin-loaded magnetic beads for 2 h, and the mixture was collected into all reaction tubes to knock down the biotin-labeled RNA complex. Afterward, beads were washed by lysis buffer five times. The RNA specifically interacting with miRNA was retrieved using TRIzol LS (Life Technologies, Thermo Fisher Scientific). Circ-ZEB1 enrichment was analyzed and evaluated by RT-qPCR and agarose gel electrophoresis (AGE), respectively.

### Fluorescence in situ hybridization (FISH)

We performed FISH on circ-ZEB1 sequence using a specific probe of miR-199a-3p-3p. Cy5-and farm-labeled probes were specific to circ-ZEB1 and miRNA, respectively. Afterward, 4',6-dimethyl-2-phenylindole (DAPI) was used to stain the nucleus. The FISH kit (GenePharma) was used in each procedure, following specific protocols. The Zeiss LSM880 NLO confocal microscope (Leica Microsystems, Mannheim, Germany) was used to acquire the images.

### Dual-luciferase reporter gene detection

The website for biological prediction (www.microRNA.org) was used to predict the binding region between PIK3CA and miR-199a-3p-3p. First, we constructed the ABCF2 30UTR gene fragment and inserted it into the pMIR-reporter (Promega, Madison, Wisconsin, USA). Next, a mutant (MUT)-binding site was designed using a complementary sequence of wild-type (WT) PIK3CA seeds. Next, the pMIR-reporter plasmid was constructed. HEK-293 T cells were co-transfected with PIK3CA-MUT or PIK3CA-WT, the luciferase reporter gene plasmid with correct sequences, with miR-199a-3p-3p mock or mock negative controls (NC) (Beinuo Life Science Co., Ltd., Shanghai, China) for 48 h. After cell collection and lysis, the luciferase activity was detected using the dual-luciferase reporter gene assay system (Promega).

### Whole-Genome sequencing

Whole-genome sequencing (WGS) is a next-generation, rapid, and cost-effective sequencing technology to determine the complete genome sequence of organisms. In other words, WGS refers to the use of high-throughput sequencing platforms for whole-genome sequencing of different individuals or groups and bioinformatic analysis at both group and individual levels. It allows comprehensive mining of DNA mutation data and offers vital data to screen tumor-causing and susceptible genes. It also helps in studying pathogenesis and genetic mechanisms. Here, WGS of LC and matched non-carcinoma tissue samples was performed to identify genes with abnormal expression in LC tissues to diagnose and treat LC.

### Animal experiment

Twelve 5-to 6-week-old female BALB/c athymic nude mice weighing 16 to 20 g were collected, with six in every group. The cells transfected with Sh-circ-ZEB1 and empty vector were injected into the right armpit of the mouse, and each nude mouse was injected with 2 × 10^5^ cells. A caliper was used to measure tumor length (L) and tumor width (W) weekly to record tumor growth, and used the following equation to calculate the tumor volume (V): V = (W ^2^ × L)/2. At week 4, following the injection, each nude mouse was sacrificed, tumor tissues were resected, and tumor weights were measured.

### Statistical analysis

Data are presented as mean ± standard deviation (SD). The GraphPad 7.0 Prism software was used for data analysis. Differences were compared by one-way analysis of variance (ANOVA) or Student’s *t*-test, followed by LSD post hoc test. When *p* < 0.05, the result indicated statistical significance. All experiments were conducted thrice.

## Data Availability

The datasets used and analyzed during the current study are available from the corresponding author on reasonable request.

## Abbreviations

Circ-ZEB1: Circular RNA zinc-finger E-box binding homeobox 1
PIK3CA: Phosphatidylinositol-4,5-bisphosphate 3-kinase catalytic subunit alpha
WGS: Whole-genome sequencing
FISH: Fluorescence in situ hybridization
HCC: Hepatocellular carcinoma
LC: Liver cancer
