# Correction: Circ-ZEB1 promotes PIK3CA expression by silencing miR-199a-3p and affects the proliferation and apoptosis of hepatocellular carcinoma

**DOI:** 10.1186/s12943-024-02036-5

**Published:** 2024-06-03

**Authors:** Weiwei Liu, Lu zheng, Rongguiyi Zhang, Ping Hou, Jiakun Wang, Linquan Wu, Jing Li

**Affiliations:** 1 Department of Hepatobiliary Surgery, the Second Affiliated Hospital of Nanchang University, 1 Mindle Road, Nanchang, Jiangxi 330006 People’s Republic of China; 2 Department of Hepatobiliary Surgery, Xinqiao Hospital, Third Military Medical University, 83 Xinqiao Main Street, Chongqing, 400000 People’s Republic of China

**Correction to: Mol Cancer 21, 72(2022)**

10.1186/s12943-022-01529-5

After the original article was published [1], The author found a typo in the subscript of the X-axis in Fig. 1B, i.e., Toumor should be in the Normal position. And in Fig. 3C the image in ShNC was used incorrectly. Therefore, the authors want to update it to the correct figures. The other elements in the figure remain the same, as does the interpretation of the results. Subsequent studies of Circ-ZEB1 by the authors confirm that the results and conclusions of this study are correct and are not affected by this erratum. The correct figures are shown below.

**Fig. 1:**
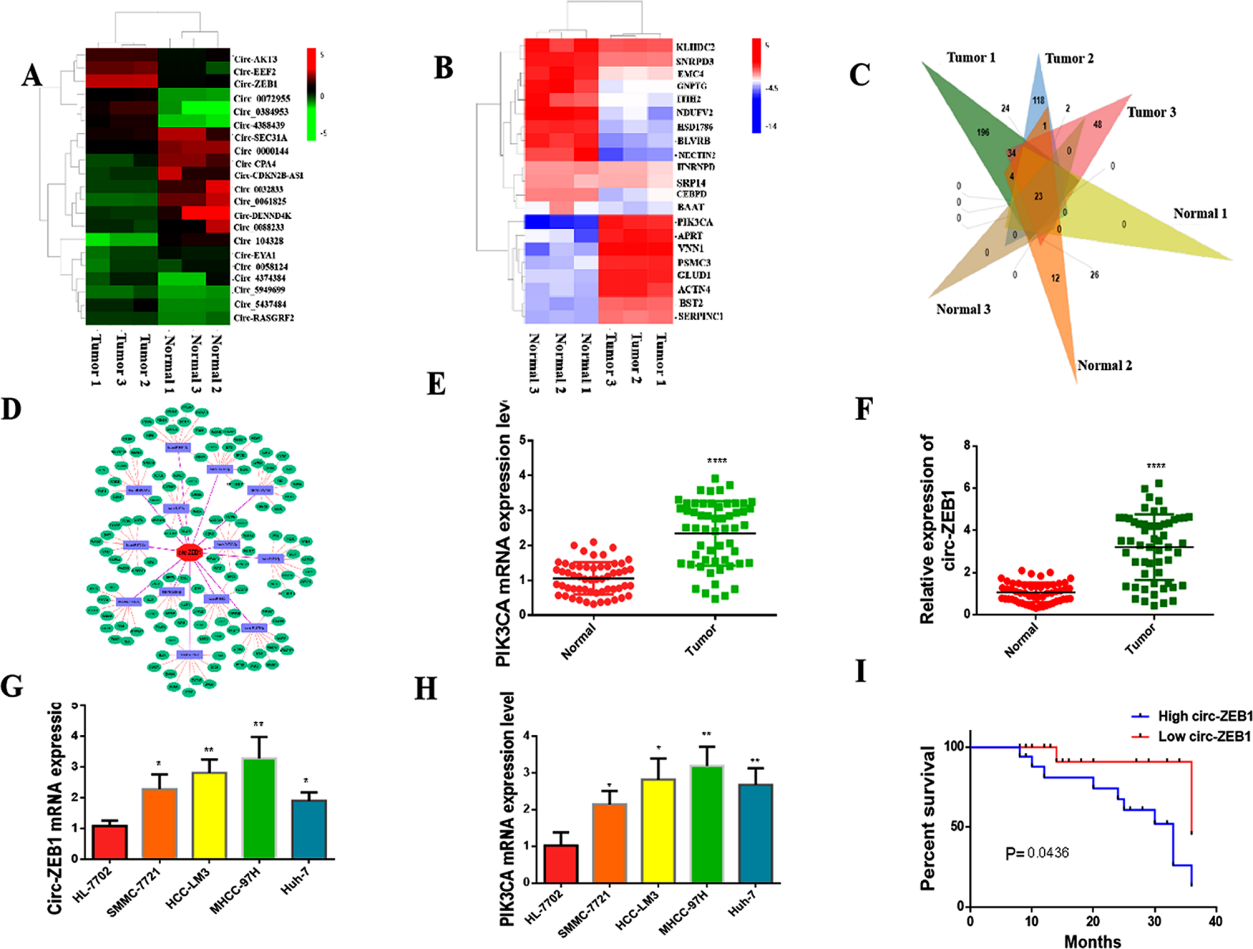
Circ-ZEB1 and PIK3CA are highly expressed in HCC. **(A)** Whole-genome sequencing revealed that circ-ZEB1 was overexpressed in HCC tissues. **(B)** Whole-genome sequencing revealed that PIK3CA was overexpressed in HCC tissues. **(C)** VENN map constructed by whole-genome sequencing. **(D)** Bioinformatics analysis revealed a relationship between circ-ZEB1 and PIK3CA. **(E)** RT-qPCR showed that circ-ZEB1 was upregulated in HCC tissues. **(F)** RT-qPCR showed that PIK3CA was upregulated in HCC tissues. **(G)** RT-qPCR showed that circ-ZEB1 was upregulated in HCC cells. **(H)** RT-qPCR showed that PIK3CA was upregulated in HCC cells. **(I)** The expression of circ-ZEB1 is correlated with the prognosis of HCC

**Fig. 3:**
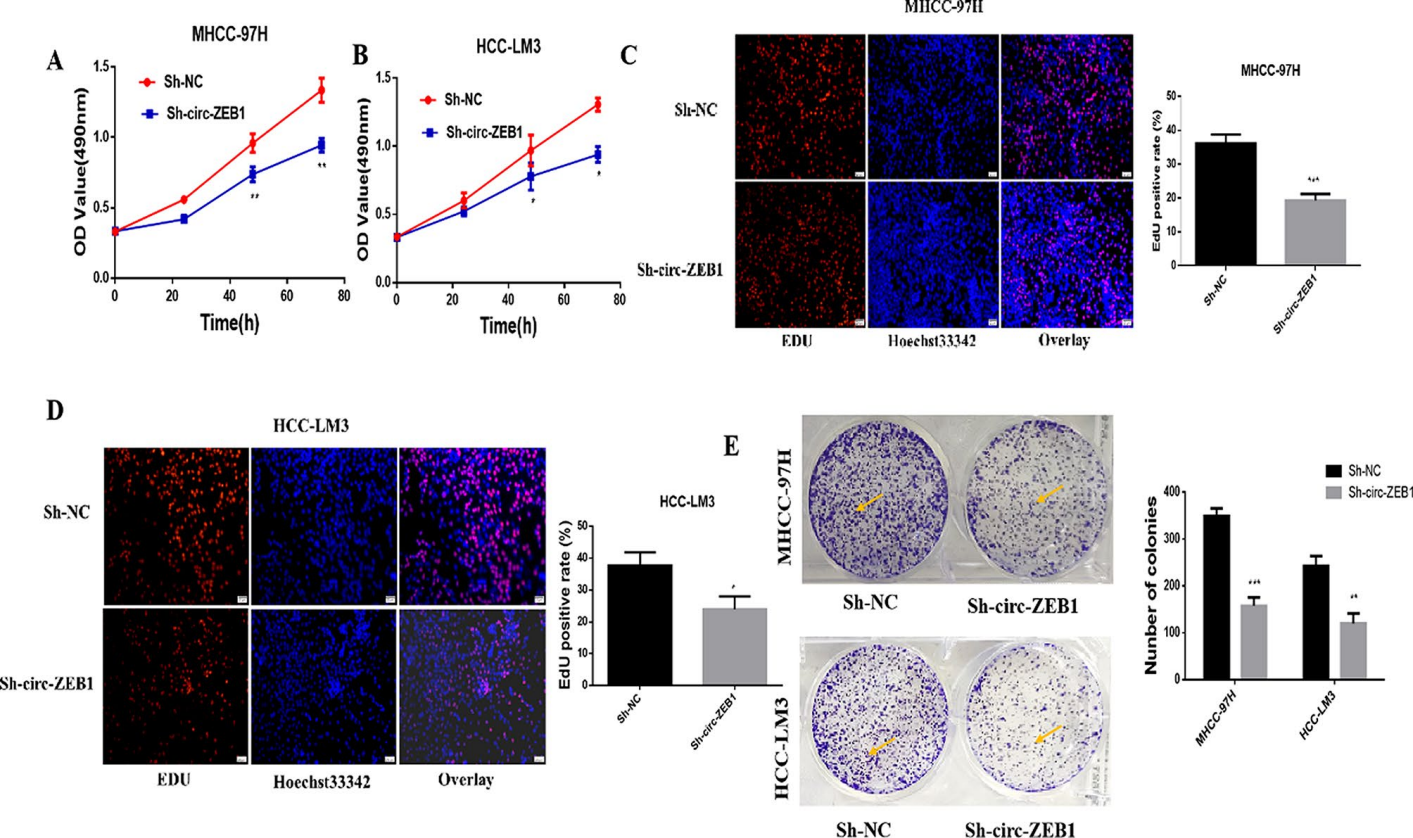
Circ-ZEB1 downregulation reduces the proliferation ability of HCC cells in vitro. **(A)** The MTT assay was used to detect the changes in the MHCC-97 H activity after the downregulation of circ-ZEB1. **(B)** The MTT assay was used to detect the changes in the HCC-LM3 activity after the downregulation of circ-ZEB1. **(C)** EdU assay reveals the changes in MHCC-97 H cell proliferation after downregulation of circ-ZEB1. **(D)** EdU assay reveals the changes in the proliferation of HCC-LM3 cells after the downregulation of circ-ZEB1. **(E)** Plate cloning experiment was used to detect cell proliferation changes after transfection of cells with Sh-circ-ZEB1. *P < 0.05 compared with the Sh-NC group. All values are presented as mean ± standard deviation (SD). Comparisons between the groups were analyzed by unpaired t-test, whereas repeated measures analysis of variance (ANOVA) was used to compare several groups. This experiment was performed thrice
